# Diabetes Education and Management in Amazon Communities

**DOI:** 10.51894/001c.164026

**Published:** 2026-07-31

**Authors:** Lauren Gesik, Rutu Patel, Suhana Posani, Oula Salih, Javier Tobar

**Affiliations:** 1 College of Osteopathic Medicine, Michigan State University

## 18

### Introduction

Diabetes mellitus is a growing public health challenge in Peru, contributing substantially to mortality and chronic complications, particularly in rural regions. Limited availability of healthcare services and patient education contribute to poor disease awareness and inadequate disease management. Using survey data, this study evaluates the status, knowledge, and self-care practices of patients with diabetes attending outpatient clinics in Iquitos, Peru. This study aims to contribute to the development of effective, culturally appropriate diabetes education programs.

### Objectives

The objective is to evaluate potential associations between demographic and lifestyle factors, diabetes control, and health literacy in the Peruvian population. We hypothesize that patients who attended the diabetes education sessions, as well as older males, and patients with lower BMI, will have better glycemic control, evidenced by lower A1C levels.

### Methods

A cross-sectional survey of 115 patients was conducted. Participants were Peruvian patients living in Iquitos and Amazon River communities enrolled in a voluntary Diabetes Clinic Program. Verbal consent was obtained from all participants. The survey collected information about self-management behaviors, diabetes-related laboratory values (A1C, glucose), health literacy, and medication data. Survey responses were coded and analyzed using SPSS.

### Results

A1C levels stratified by sex show slight differences but are not statistically significant. Females registered higher A1Cs than males with t-tests values for mean differences (A1C of 9.33 and 7.73) significant at a *p* < 0.06 level. Comparison of glucose levels showed similar results. Correlation between age and A1C (Pearson correlation of -0.37, *p* < 0.05) shows that with age patients have better A1C control. Mean A1C levels for patients who attended previous education sessions were not statistically significant compared to those who did not. However, the small differences detected became significant with increased sample size.

### Discussion

The A1C and BMI differences between sexes are likely due to differences in occupation-related physical activity. Given the small sample size, the observed age-related differences in A1C may be attributed to life expectancy of patients. Extrapolated data suggests that prior education and being male correlate with better glycemic control. A larger sample could provide more evidence about the effect of diabetes education.

